# Gradient coating of extracellular matrix derived from endothelial cells on aligned PCL nanofibers for rapid endothelialization

**DOI:** 10.3389/fbioe.2024.1527046

**Published:** 2025-01-08

**Authors:** Ziyi Zhou, Yijing Lin, Na Liu, Yiming Zhang, Bing Li, Yuanfei Wang

**Affiliations:** ^1^ Qingdao Medical College, Qingdao University, Qingdao, China; ^2^ Department of Plastic, Reconstructive and Cosmetic Surgery, Xinqiao Hospital, Army Medical University, Chongqing, China; ^3^ Department of Genetics and Cell Biology, Basic Medical College, Qingdao University, Qingdao, China; ^4^ Central Laboratory, Qingdao Stomatological Hospital Affiliated to Qingdao University, Qingdao University, Qingdao, China

**Keywords:** vascular scaffold, aligned nanofibers, endothelial cells, cell migration, endothelization

## Abstract

**Introduction:**

Artificial vascular scaffolds can mimic the structure of natural blood vessels and replace the damaged vessels by implanting them at the injury site to perform the corresponding functions. Electrospinning technology can perfectly combine biological signals and topographical cues to synergistically induce directed cell migration and growth.

**Methods:**

In this study, poly (caprolactone) (PCL) nanofibers, PCL nanofibers uniformly coated with the extracellular matrix derived from endothelial cells (ECd), and bi-directional linear gradient ECd-coated PCL nanofibers were prepared by electrospinning and electrospray techniques to evaluate their effects on the proliferation and migration of Human umbilical vein endothelial cells (HUVECs) and rapid endothelialization.

**Results:**

The results showed that HUVECs could successfully adhere to the surface of these three nanofibers and maintain high viability. The migration results indicated that the bidirectional linear gradient coating could accelerate the migration of HUVECs and the endothelialization process. On this basis, three types of bionic vascular scaffolds, including PCL vascular scaffold, uniform ECd-coated PCL vascular scaffold, and bi-directional linear gradient ECd-coated PCL vascular scaffold, were further prepared. The results showed that the topology and biological signal of the bi-directional linear gradient ECd-coated PCL vascular scaffold synergistically promoted the migration of HUVECs more effectively.

**Discussion:**

This provides a new way to clinically promote the structural and functional recovery of damaged vessels and develop personalized or universal artificial vascular scaffolds, which is of great importance in cardiovascular regenerative medicine.

## 1 Introduction

Cardiovascular disease (CVD) is one of the most common diseases in the world, characterized by high pathogenicity and mortality (Vong et al., 2018; Wang et al., 2022a; Qian et al., 2021). The clinical treatment of CVD consists of three main approaches: pharmacotherapy, which is the most carried out treatment modality and the basis of CVD treatment; interventional treatments, including radiofrequency ablation and cardiac pacing therapy; and surgical treatments, including bypass therapy and cardiovascular transplantation (Abdelsayed et al., 2022; Lunyera et al., 2023; Krahn et al., 2018). Vascular grafts are primarily used to restore or establish new blood flow pathways to maintain or improve circulation to an area of tissue or organ, such as in cases of defective vascular segments due to trauma or resection or arterial embolism or lymphatic obstruction requiring a “bypass” to form a circulatory system (Xing et al., 2021; Zhao et al., 2023). Vascular grafting requires the supply vessel to have the same outer diameter and sufficient length as the recipient’s vessel. The graft also faces problems such as impaired circulation (ischemia or stasis) in the donor area. Therefore, there is an urgent need for high-performance artificial vascular grafts to replace autologous vessels for blood flow reconstruction. Currently, small-diameter artificial vessels (<6 mm) are mainly used in coronary artery bypass grafting, peripheral vascular bypass grafting, vascular trauma (defects ≥2 cm), tissue vascular access for hemodialysis, and restoration of organ function (Asakura et al., 2019; Wang et al., 2021; Wu et al., 2018). However, artificial vascular grafts can lead to serious complications, such as thrombosis and endothelial hyperplasia at the anastomosis site, impairing lumen patency (Oliveira et al., 2020; Teebken and Haverich, 2002; Zhuang et al., 2020). In addition, although the current artificial blood vessel stents have certain mechanical properties and biocompatibility or can provide biochemical signals required for vascular regeneration, there are still obvious shortcomings in simulating the structure and function of natural blood vessels. Existing stents are often unable to simulate the topology of natural vascular networks adequately and induce cell crawling, thus affecting the effectiveness of vascular stents in clinical applications (Liang et al., 2016; Cheng et al., 2022). Therefore, to improve the patency of small-diameter artificial vessels, improving biocompatibility/endothelialization/mechanical properties through material selection and surface modification has become a key research direction.

Electrospinning technology can prepare micro/nanofibers with high specific surface area and porosity, which can mimic the extracellular matrix, promote cell adhesion, proliferation, and differentiation, and provide a good growth environment for cells. The design of the receiver device allows the preparation of tubular structures with different diameters and is, therefore, an ideal method for the preparation of small-diameter artificial vascular scaffolds (Yao et al., 2022; Guo et al., 2023; Song et al., 2023; Wang et al., 2022b). In particular, the vascular scaffolds prepared by this technology can be loaded with biological factors, thus improving the biocompatibility of vascular scaffolds and promoting rapid endothelialization of blood vessels. Although current artificial vascular scaffolds already have certain mechanical properties, biocompatibility or can provide biochemical signals required for vascular regeneration, how to combine the advantages of existing vascular scaffolds and synergize the topology of biomaterials and biochemical cues to build tools that are beneficial for cardiovascular therapy is still a difficult and hot topic for clinical trial research (Wei et al., 2021; Liu et al., 2023a; Chen et al., 2023).

Vascular endothelial cells are highly active cells that maintain vascular tone and structure by mediating the balance of vasodilation, contraction, growth inhibition, growth promotion, anti-inflammation, and pro-inflammation. Endothelial progenitor cells have been reported to differentiate into phenotypically and functionally mature endothelial cells capable of synthesizing and secreting various bioactive substances, thereby maintaining long-term vascular patency (Zhang et al., 2022). Endothelialization of the material surface reduces thrombus formation, and the endothelial tissue on the vascular surface is a natural anticoagulant tissue containing natural anticoagulant components such as heparin, prostaglandins, and nitric oxide (Brown et al., 2013). The migration of endothelial cells is closely related to the rapid endothelialization of blood vessels. It has been shown that various growth factors and cytokines secreted by vascular endothelial cells can induce endothelial cell proliferation and migration. Therefore, future research focuses on how to effectively load endothelial cell secretions onto artificial vascular scaffolds by electrospinning technology.

Many studies have been conducted to promote cell proliferation, targeted migration, and rapid endothelialization by optimizing the topology of artificial vessels, biochemical cues, and synergistic regulation of both. In this study, we developed a novel artificial vascular scaffold. First, ECd was coated on the surface of aligned PCL nanofibers in a bi-directional linear gradient, and relevant experiments confirmed its ability to promote the proliferation and migration of HUVECs and rapid endothelialization. Further, we investigated the migration of HUVECs on PCL nanofiber tubes with bi-directional linear gradient coating of ECd to evaluate its role in promoting vascular regeneration and biosafety.

## 2 Materials and methods

### 2.1 Materials

Porcine skin type I gelatin, PCL and rhodamine B, were purchased from Sigma-Aldrich (America). 1,1,1,3,3,3-hexafluoro-2-propanol (HFIP) was purchased from Macklin (Shanghai, China). Phalloidin-iFluor 488 and anti-CD31 were purchased from Abcam (Shanghai, China). 4′,6-diamidino-2-phenylindole (DAPI) were bought from Solarbio (Beijing, China). Triton X-100, bovine serum albumin (BSA), and Tween-20 were products of Solarbio (Beijing, China). CCK-8 assay kit was bought from NCM Biotech (Suzhou, China). Dulbecco’s modified eagle medium (DMEM) and antibiotic-antimycotic were purchased from Solarbio (Beijing, China). Fetal bovine serum (FBS) was purchased from Pan (Germany).

### 2.2 Collection of conditioned culture medium to obtain the ECd

1 × 10^5^ HUVECs were taken and cultured for 3, 5, and 7 days. 2 × 10^5^ HUVECs were cultured for 3 days, and the cells were cultured in a complete medium containing 1% FBS, and the conditioned medium was collected. The effect of ECd proteins on HUVECs proliferation in the culture medium at different times and concentrations was examined with a CCK-8 kit, and the optimal action concentration and time were finally determined. High sugar medium containing 10% FBS was mixed with a conditioned medium at a ratio of 1 : 1. HUVECs were seeded in 24-well plates at a density of 5 × 10^3^ cells/mL, supplemented with the above mixed medium, incubated at 37°C and in an incubator containing 5% CO_2_, and the viability of HUVECs was determined within 1, 3, and 5 days. The absorbance of 450 nm were measured using an enzyme marker (MultiskanMK3, Thermo, United States). After determining the parameters, we collected a conditioned medium and freeze-dried it to obtain solid ECd.

### 2.3 Fabrication and characterization aligned PCL nanofibers

Aligned PCL nanofiber scaffolds were prepared by electrospinning 10% PCL dissolved in HFIP using a high-speed drum (3,000 rpm) with 10 kV high voltage, 1.0 mL/h flow rate, and 15 cm collecting distance. The nanofiber scaffolds were dried in a negative pressure vacuum for 48 h. Then, the scaffolds were fixed on blank square slides (20 mm × 20 mm). The nanofiber scaffolds were then immersed in PDL solution at 4°C and prepared for use. Scanning electron microscopy (SEM) (S-4800, Hitachi, Japan) was used to examine the morphology of the scaffolds. ImageJ was used to examine the diameter length of the fibers.

### 2.4 Fabrication and characterization of the bi-directional linear ECd gradient

40 mg ECd was weighed with 20 mg gelatin and dissolved by 400 μL ddH_2_O. Then, 1,600 μL acetic acid was added and stirred on a magnetic stirrer for 2 h. To demonstrate the gradient formation, 10 mg of rhodamine B was added to the solution, and the gradient formation was determined based on the fluorescence intensity. Based on previous studies, we used a special electrospraying method to prepare bioactive protein gradients (Zhou et al., 2022). To generate ECd bi-directional linear gradients on square glass slides (20 mm × 20 mm), strip magnets of different widths (4 mm, 8 mm, 12 mm, 16 mm) were placed under the glass slides with their edges aligned with the edges of the square slides. The last layer of ejected ECd droplets was collected without magnets. We used a mask with a slit in the middle in each process to facilitate collection. The mask was the same size as the underneath magnet. For electrospraying, the flow rate was 0.4 mL/h, the voltage was 25 kV, and the receiving distance was 15 cm. The relative fluorescence intensity of different gradients on the glass slides was measured with a fluorescence microscope and the ImageJ software. In addition, the hydrophilicity of aligned PCL nanofiber and ECd-coated PCL nanofibers was investigated using the CA system (OCA20).

### 2.5 HUVECs proliferation and migration

To examine the proliferation of HUVECs on the different nanofiber scaffolds after culturing for 1, 3, and 5 days, the control group and bare aligned PCL nanofiber scaffold (blank) were used as control. 1 × 10^4^ cells/mL were cultured in each well. At each time, the cells were washed with PBS, and then fresh medium containing 10% CCK-8 reagent was added to each well. After 3 h, the supernatant at 450 nm was measured. After 3 days of incubation, we stained the cells on the different nanofiber scaffolds with a live/dead staining kit to observe the survival of HUVECs in different groups.

Subsequently, we investigated the effect of ECd bi-directional linear gradient and aligned nanofiber scaffolds on HUVECs migration. The aligned PCL nanofiber scaffolds with gradient and uniform ECd coating and blank nanofiber scaffolds were transferred to 6-well plates, respectively. A 10 mm × 20 mm rectangular mold of polydimethylsiloxane (PDMS) was placed in the center of the samples. HUVECs were inoculated bilaterally at the bare regions left by the PDMS column at a concentration of 1 × 10^5^ cells/mL. After cell adhesion, the PDMS was removed, allowing the cells to migrate freely.

After 3 days of incubation, AlexaFluor 488 and DAPI staining of different samples were performed to detect cell migration. Briefly, cells were fixed in 4% paraformaldehyde solution for 10 min at room temperature and permeabilized with 0.1% Triton X-100 for 10 min 1% BSA was used to reduce non-specific background staining, and samples were incubated in the staining solution for 20 min. Afterward, nuclei were stained with DAPI for 5 min and washed 3 times with PBS. After staining, the samples were imaged with a Nikon orthomosaic microscope. The entire migration zone was divided equally into three regions. Using the ImageJ software, the number of cells in each migration zone was measured based on the DAPI-stained fluorescence micrographs. The morphology of cells in zone II was observed under magnification.

### 2.6 HUVECs viability and immunostaining

We first tested the viability of HUVECs on the different types of nanofiber scaffolds. HUVECs were inoculated on the different scaffolds for 3 days and then assayed using CCK-8. The pure glass and blank groups were used as control groups. In this case, the amount of FBS was changed from 10% to 1% in the culture medium. At 3 days, the samples were fixed overnight at 4°C in 4% paraformaldehyde, incubated with CD31 primary antibody (1 : 200, ABclonal) alone overnight at 4°C, and stained with Alexa fluor 488-coupled secondary antibody (1 : 1,000, ABclonal) for 2 h at room temperature before observation.

### 2.7 Rolling the different nanofibers to obtain vascular scaffolds

After collecting the PCL nanofibers continuously for 6 h, with a uniform and bi-directional gradient, ECd was separately coated on the nanofibers. The blank PCL nanofibers served as the control. To obtain the vascular scaffolds, the nanofiber mats were rolled into tubes, and a bio-gel sealed the edges. The stereoscopic images of the vascular scaffolds were taken by ultra-deep three-view digital microscopy.

### 2.8 HUVECs migration on the lumen of different vascular scaffolds

HUVECs were seeded at both ends of the vascular scaffolds (*i.e.*, PCL vascular scaffold, uniform ECd-coated PCL vascular scaffold, and bi-directional linear gradient ECd-coated PCL vascular scaffold), and the distribution and migration of HUVECs were observed. The tubes were cut into 1 cm in length and placed in 24-well plates. After soaking the tubes in complete medium for 2 h at 37°C, HUVECs were seeded at a density of 1.5 × 10^5^ cells/mL, and the medium was renewed every 2 days. After culturing for 7 days, the samples were fixed with 4% paraformaldehyde for 20 min. Then, cross-sectional and longitudinal frozen sections were separately obtained for staining with CD31 and DAPI. The distribution of cells was observed under the fluorescent microscope.

### 2.9 Statistical analysis

Results are presented as mean ± standard deviation (SD). Statistical comparisons between groups were performed using one-way ANOVA, followed by Student’s t-tests for all pairwise comparisons. *P < 0.05, **P < 0.01, and ***P < 0.001 were considered statistically significant.

## 3 Results and discussion

### 3.1 Effects of ECd on HUVECs proliferation

ECd secreted by different numbers of cells at different time points were collected and freeze-dried. Supplementary Figure S1 shows the proliferation of HUVECs on days 1, 3, and 5 of culture. On day 1, there was little difference in cell proliferation between groups, and on days 3 and 5, the ECd collected from the 1 × 10^5^ cell count group was significantly higher than that of other groups, this may be due to the excessive secretion of ECd (Lee et al., 2022). The above results suggested that ECd generated by appropriate cell numbers and culture time can accelerate cell proliferation effectively.

### 3.2 Generation of ECd bi-directional linear gradient on aligned PCL nanofibers

**FIGURE 1:**
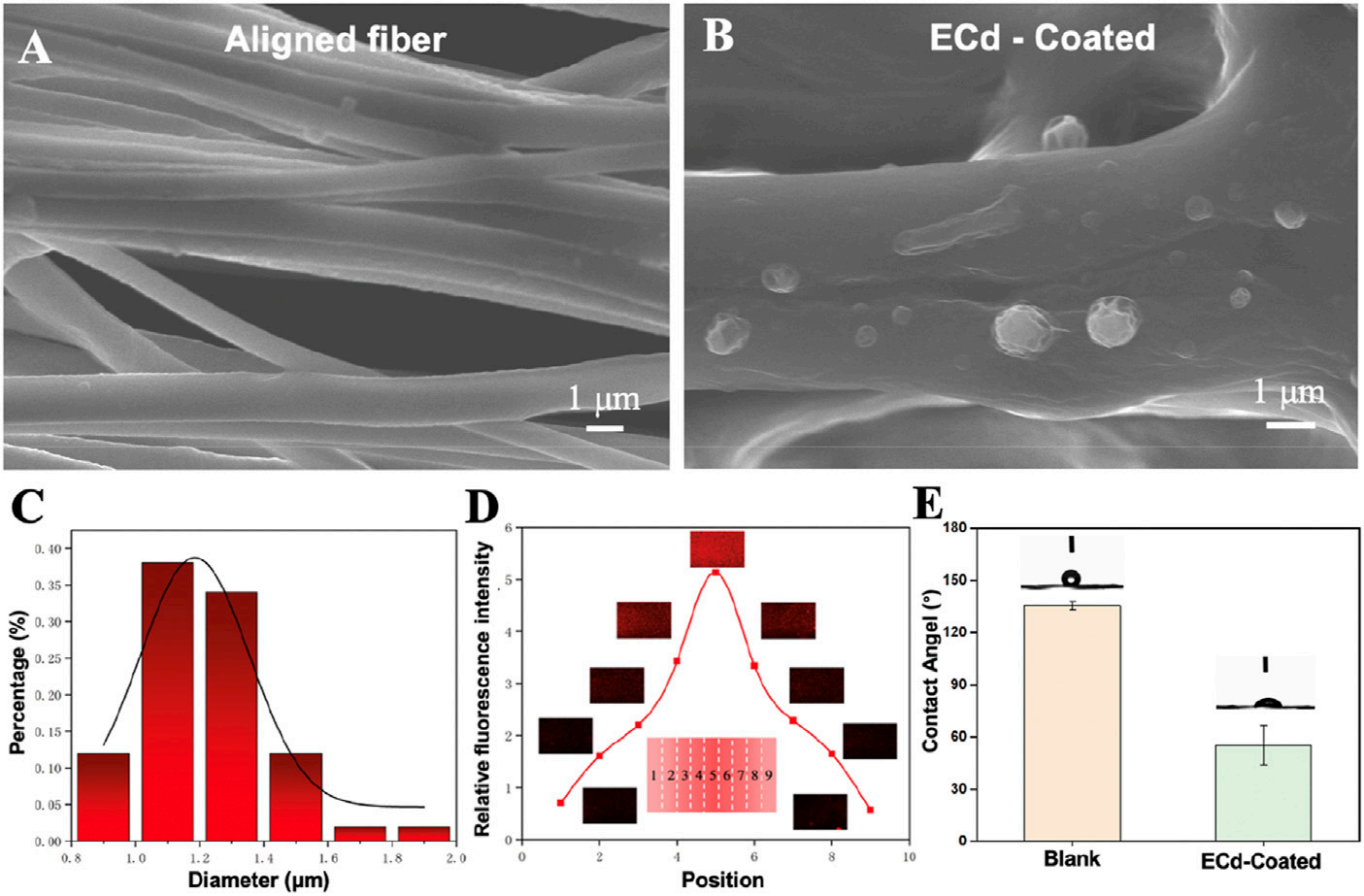
**(A)** SEM images of aligned PCL nanofibers. **(B)** SEM images showing the electrospray particles of ECd on the aligned PCL nanofiber scaffolds. **(C)** Fiber diameter distribution (n = 50). **(D)** Relative fluorescence intensity and corresponding fluorescence micrographs showing the bi-directional linear gradient of ECd containing rhodamine B. **(E)** Blank and ECd-coated blank contact angel.

Biofunctionalized vascular scaffolds were prepared by electrospinning and electrospraying techniques. As shown in Figures 1A, C, aligned nanofibers with a diameter of approximately 800 nm ± 100 nm were obtained, with uniform fiber distribution and distinct orientation. It was shown that the highly aligned electrospun nanofibers could accelerate the regeneration of defective tissues (Tindell et al., 2023; Yeo and Kim, 2019; Yeo and Kim, 2018). Before electrospraying ECd, the vascular scaffold was coated with poly-d-lysine, leaving cationic sites to bind to protein sites (Ji et al., 2019). According to previous studies, it was confirmed that protein-like substances tend to adsorb as a monolayer on the surface of poly-d-lysine-treated materials. Also, we reported a method to generate a bi-directional linear gradient of active proteins on a planar surface by the electrostatic field distribution during electrohydrodynamic jet printing, where particles ejected from the nozzle are deposited on the substrate by a special device with a particle density proportional to the deposition time (Zhou et al., 2022). Thus, by varying the deposition time, a particle density gradient can be generated on the substrate (Yan et al., 2018). As shown in Figure 1B, ECd particles were deposited on aligned nanofiber scaffolds, and cell migration was induced by preparing this bi-directional gradient ECd particles, thus providing biochemical signaling guidance to the cells.

Surface-modified bioactive substances with inducing properties of materials must be specifically designed and functionalized for the desired target tissue repair (Liu et al., 2023b; Fraioli et al., 2016). In a previous study, MNP-TGF/bFGF-PLGA scaffolds mimicking the physical and biochemical characteristics of natural endoplasmic periplasmic media were prepared using soft light microscopy combined with solution casting and phase separation techniques. Under the synergistic effect of dual surface morphology and release growth factors, MNP-TGF/bFGF-PLGA scaffolds have good phenotypic modulation of vascular smooth muscle cells (Shen et al., 2016). Therefore, the ideal tissue-engineered vascular scaffold should mimic natural blood vessels’ physical and biochemical cues to guide cell growth and tissue formation. To fully demonstrate the feasibility of the present method, we labeled the obtained ECd bi-directional gradients with rhodamine B during the electrospraying process. Figure 1D shows the fluorescence micrographs of the particle density of successive gradients at different sample locations. The gradient showed a trend of increasing and then decreasing after reaching the highest point, with a 5-fold difference between the highest and lowest gradients. The results show that we successfully prepared a bi-directional linear continuous gradient of ECd on aligned PCL nanofibers. Figure 1E shows that the fiber film without ECd coating has hydrophobicity, but after ECd coating, it can be found that the hydrophilicity of the fiber scaffold can be enhanced, and this pure natural substance coating can promote cell adhesion.

### 3.3 Proliferation and migration of HUVECs on aligned PCL nanofibers coated with ECd bi-directional linear gradient

**FIGURE 2:**
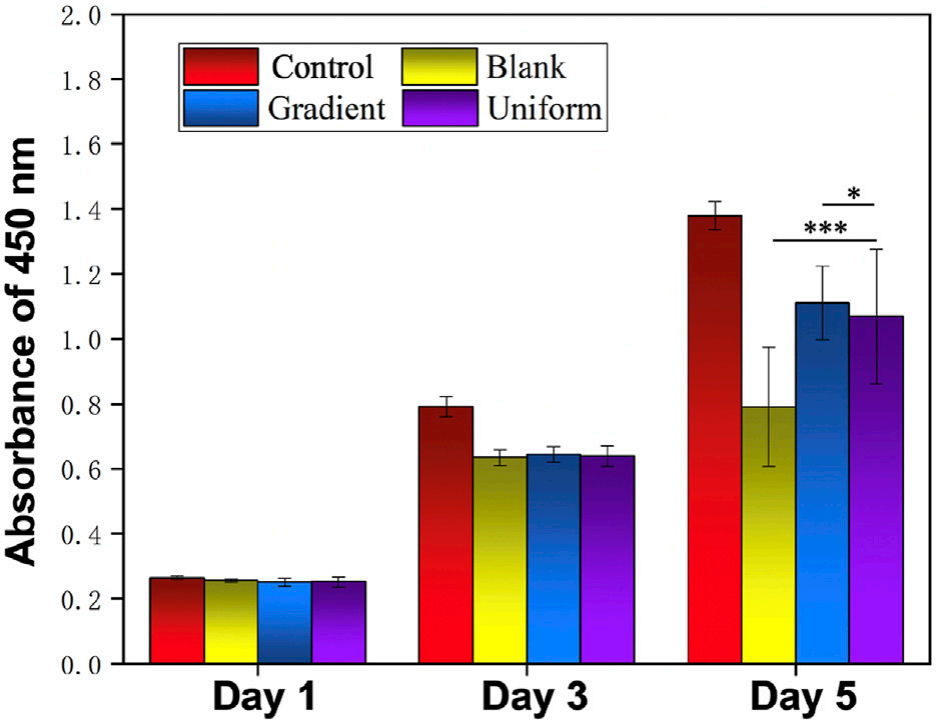
Proliferation of HUVECs on blank PCL nanofibers, PCL nanofibers coated with uniform and gradient ECd, and control group after culturing for 1, 3, and 5 days. ***P < 0.001 as compared with PCL nanofibers coated with gradient and uniform ECd, *P < 0.05 as compared with PCL nanofibers coated with gradient ECd.

**FIGURE 3:**
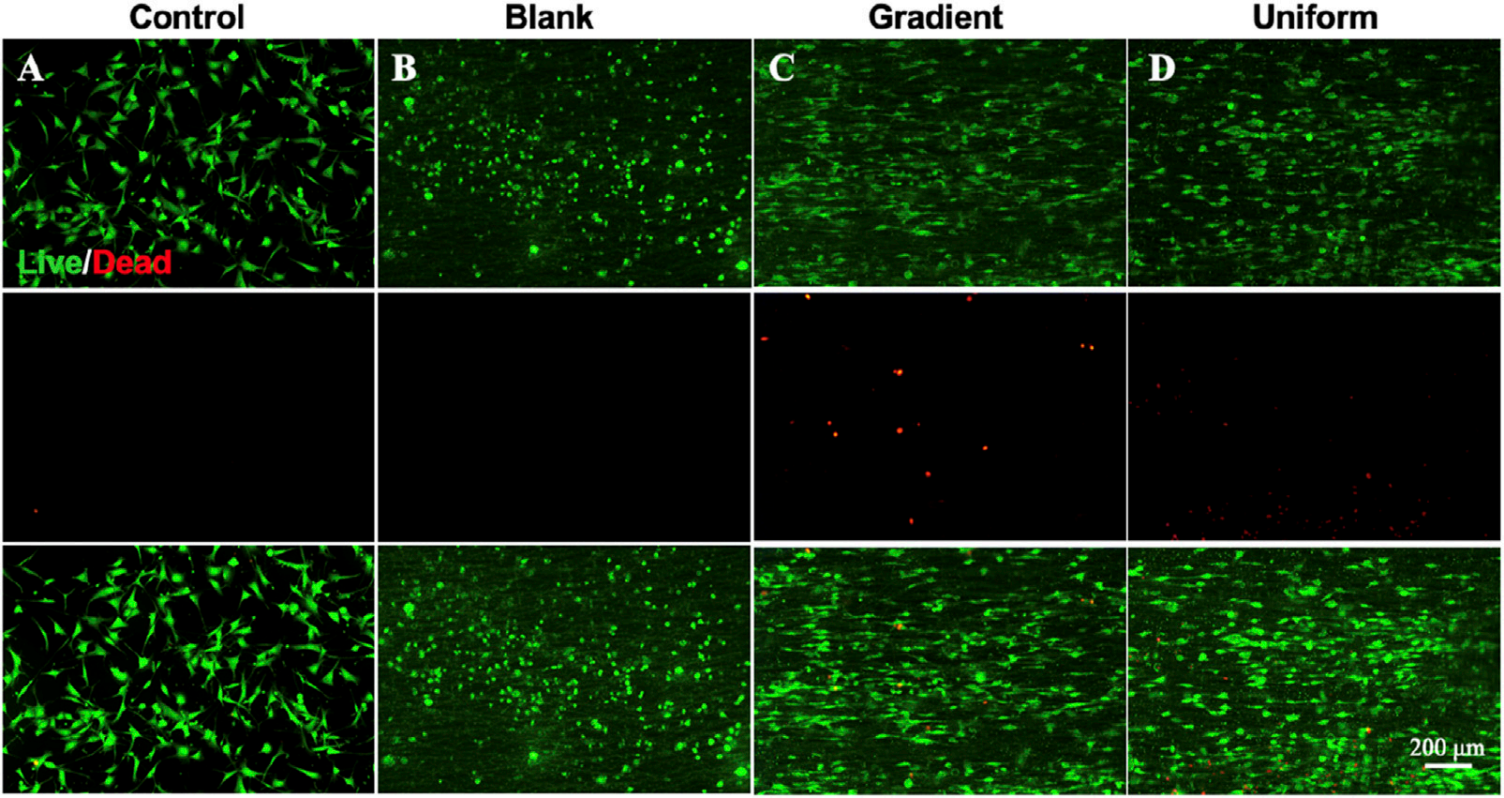
Live and dead staining micrographs showing the survival situation of HUVECs on **(A)** control, **(B)** blank PCL nanofibers, PCL nanofibers coated with **(C)** gradient, and **(D)** uniform ECd after culturing for 3 days.

In order to investigate the synergistic effect of the bi-directional linear gradient of aligned PCL nanofiber scaffolds and ECd particles on HUVECs migration and proliferation, we prepared aligned PCL nanofiber scaffolds encapsulated with ECd gradient particles to compare them. We first investigated the proliferation of cells on three different types of scaffolds using pure glass and pure aligned PCL nanofiber scaffolds as control groups. As shown in Figure 2, on days 1 and 3, there was no significant difference between the gradient and uniform groups compared to the control group (*P* > 0.05). On day 5, there was a small significant difference between the gradient and uniform coating groups. Therefore, no matter what kind of ECd coating has the effect of promoting HUVECs proliferation. In addition, cell proliferation on either ECd bi-directional linear gradient or uniform covered scaffolds was superior to that of aligned PCL nanofiber scaffolds (*P* < 0.001). Wrapping nanofiber scaffolds with ECd particles improved cell proliferation and viability, mainly due to the nutrients in ECd that accelerate cell migration, proliferation, and angiogenesis (Castelli et al., 2016; Luo et al., 2018). The results of live and dead staining (Figures 3A–D) were consistent with CCK-8. The materials in each group showed no cytotoxicity, and it was found that the HUVECs growing on the aligned PCL nanofibers showed a “long strip”, crawling along the fiber, in which the number of cells in the gradient group and the uniform group was higher than those in the other two groups. The aligned PCL nanofiber scaffolds significantly influence cell behavior, particularly when the fibers align with the direction of cell migration. This indicates that the orientation of the fibers offers physical guidance for cell migration. Using aligned PCL nanofibers along with the bi-directional linear gradient of ECd can further enhance the cell migration effect, as both provide induction signals that promote migration in a specific direction.

**FIGURE 4:**
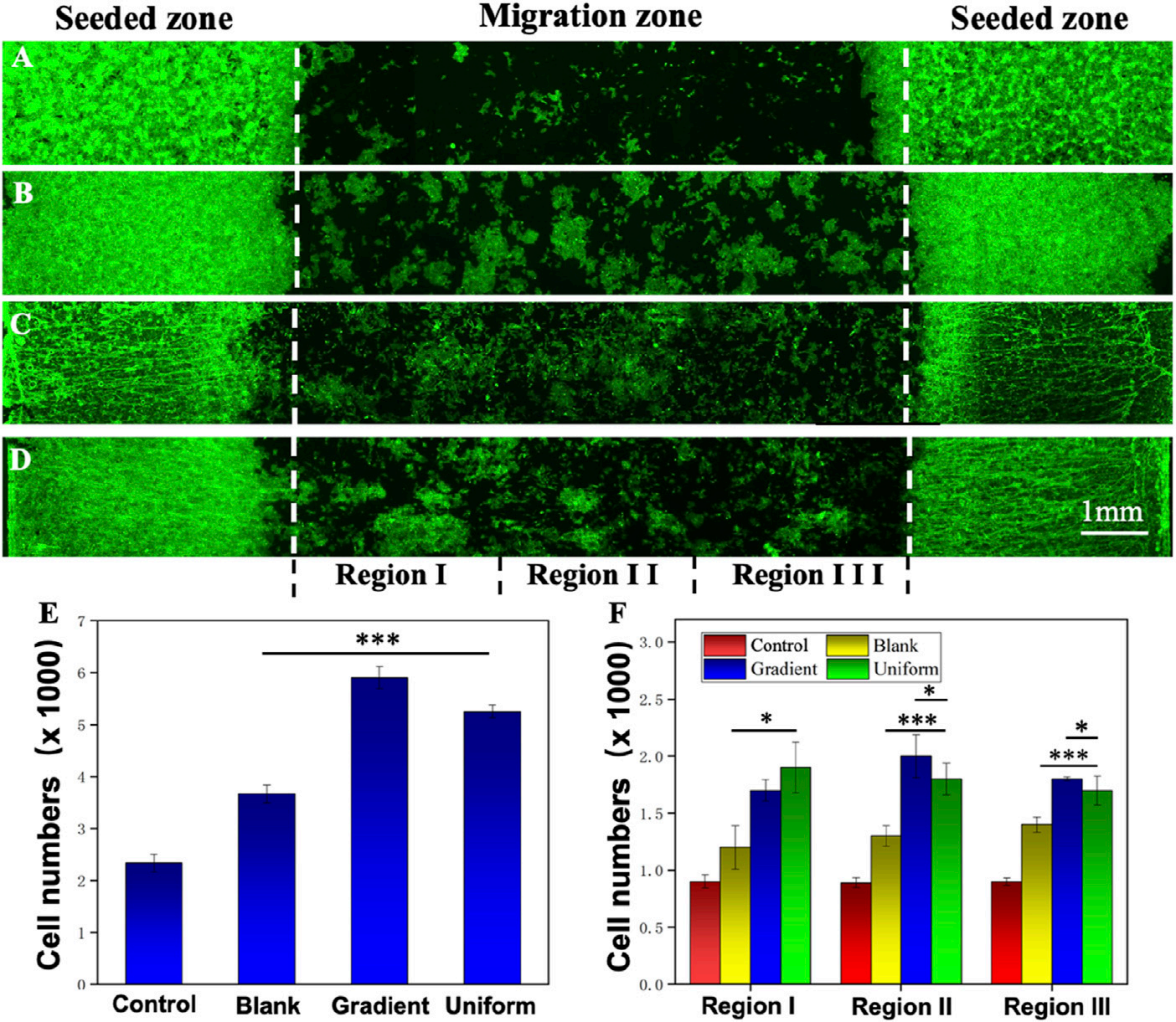
Fluorescence micrographs showing the migration of HUVECs on **(A)** control, **(B)** blank PCL nanofibers, PCL nanofibers coated with **(C)** gradient, and **(D)** uniform ECd after culturing for 3 days. **(E)** Total number of HUVECs in the migration zone. **(F)** The number of migrated HUVECs in different areas of the migration zone. Regions I, II, and III are referred to 1, 2, and 3 in **(A–C)**. ***P < 0.001 compared with PCL nanofibers coated with gradient and uniform ECd, *P < 0.05 compared with PCL nanofibers coated with gradient ECd.

The migration of HUVECs on different nanofiber scaffolds were studied using specific cell migration methods with glass and aligned PCL nanofiber scaffolds as controls. After 3 days of incubation, the cells were stained with DAPI and Alexa Fluor 488 phalloidin to observe cell migration on different samples. Figures 4A–D shows the route of cell migration of HUVECs on four different samples. On the aligned PCL nanofiber scaffolds coated with ECd bi-directional linear gradient (Figure 4C), the cells covered almost the entire migration zone, with many cells visible in the center. The physical cues provided mainly by the aligned PCL nanofiber scaffolds and the ECd bi-directional linear gradient synergistically promote the migration of cells toward the direction of increased ECd content. Meanwhile, the quantitative plots of the total migrating cell count and the number of migrating cells within the sub-region in Figures 4E, F again validated the migration results. The total number of cells in the gradient group was higher than in the uniform group (*P* < 0.001), possibly because the cells migrated rapidly from places with low protein concentrations to places with high protein concentrations. In contrast, cells on scaffolds consisting of ECd uniform coated encapsulated aligned PCL nanofiber and bare PCL nanofibers (Figures 4B, D) had more cells in the migration zone than in the migration zone of pure glass (Figure 4A), suggesting that the contact-guided cues provided by nanofiber scaffolds alone can also accelerate the induction of cell migration. More cells were in the pure control I area and fewer in the central area, indicating that the cells were not driven to migrate. The cytoskeleton, consisting of actin microfilaments, microtubules, and intermediate filaments, has been reported to play a key role in cell migration (Blanchoin et al., 2014; Mayor and Etienne-Manneville, 2016). The bi-directional linear gradient of ECd can accelerate the migration and endothelialization of HUVECs. This accelerated effect may be attributed to the physical and chemical signals formed by the gradient, which act synergistically on the cells to guide their migration toward regions with higher ECd content. ECd, as a bioactive substance containing a variety of glycoproteins, can enhance cell viability and proliferation capacity while promoting angiogenesis, and these properties may also indirectly facilitate HUVECs’ migration along the gradient coating.

As shown in Supplementary Figure S2B–D, the cytoskeleton grows on aligned nanofibers, with actin microfilaments evident. It has been shown that the surface morphology (presented density and size) and biochemical environment of biomaterials strongly influence cell behavior and a highly cell-type-specific response to the range and scale of morphological features (Faia-Torres et al., 2014; Yang et al., 2017). Thus, cells perceive the induction provided by aligned PCL nanofibers and the ECd bi-directional linear gradient migrating towards the central region with the highest ECd content.

### 3.4 Cell viability and CD31 presentation of HUVECs on aligned PCL nanofibers coated with ECd bi-directional linear gradient

**FIGURE 5:**
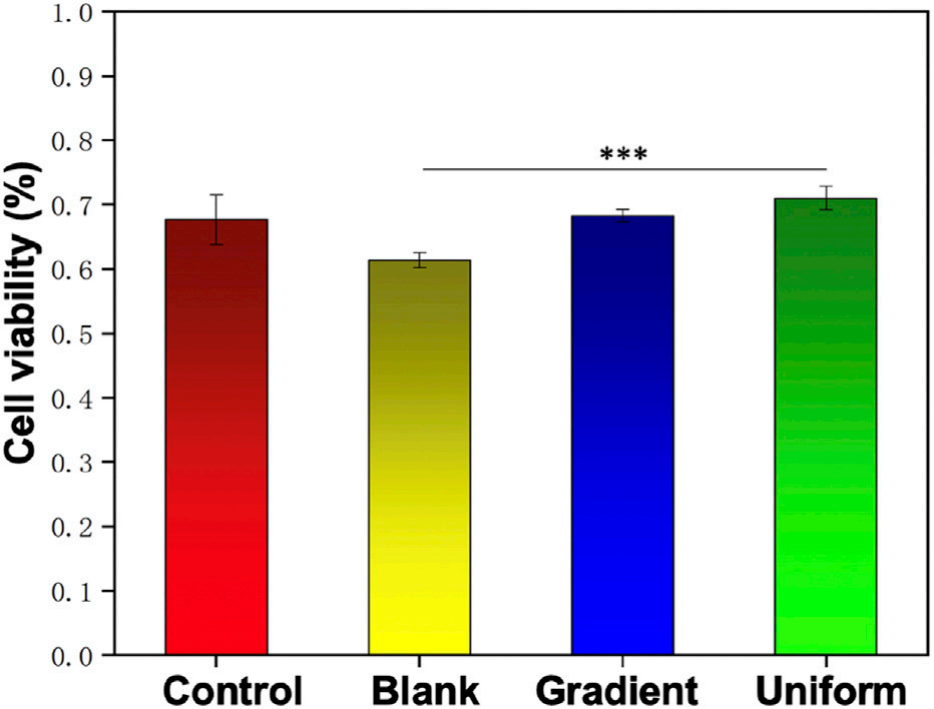
The viability of HUVECs on the control group, blank PCL nanofibers, and PCL nanofibers coated with gradient and uniform ECd after culturing for 3 days. ***P < 0.001 compared to the PCL nanofibers coated with gradient and uniform ECd.

The activity of HUVECs on ECd coated PCL nanofiber scaffolds were evaluated by CCK-8 reagent kit, and pure glass and blank aligned PCL nanofiber scaffolds were used as control groups. Figure 5 shows the viability of HUVECs on day 3 of culture on different nanofiber scaffolds, the cells were significantly more active on aligned PCL nanofiber scaffolds with gradient and uniform ECd coated than control and blank. The results showed that the nanofiber scaffolds with ECd bi-directional bilinear gradient coated could further improve the cellular activity of HUVECs, mainly because ECd is a non-cellular 3D macromolecular network composed of several glycoproteins such as collagen, proteoglycan, elastin, fibronectin, and laminin, which can improve cell viability and proliferation and angiogenesis (Ju et al., 2022).

**FIGURE 6:**
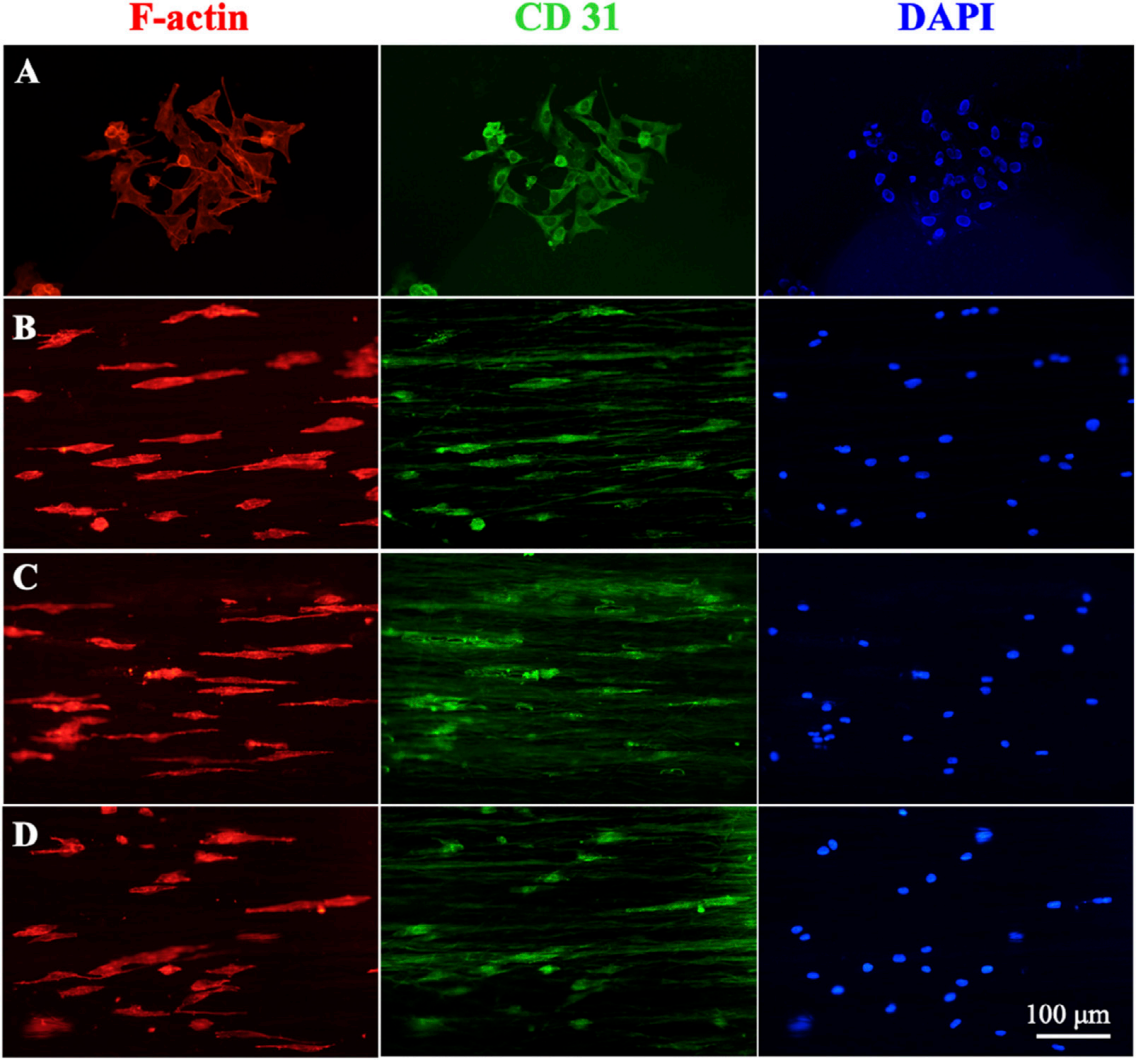
Immunofluorescence staining micrographs showing the HUVECs after culturing on **(A)** control, **(B)** blank PCL nanofibers, PCL nanofibers coated with **(C)** gradient, and **(D)** uniform ECd after culturing for 3 days. Red: F-actin; Green: CD31; Blue: DAPI.

Figure 6 shows the cell morphology and CD31 expression of HUVECs cultured on different nanofiber scaffolds. Figures 6B–D showed that HUVECs were extended in the aligned PCL nanofiber cell morphology, while the cells in the blank group were spindle shaped. This arrangement of aligned nanofibers were conducive to HUVECs crawling along the fibers and accelerating cell migration. We also found that CD31 was expressed in both the blank group and the gradient or uniform group, indicating that the manufacture of such vascular scaffolds can better promote vascular endothelealization, and the gradient coating of ECd can accelerate the generation and migration of cells in blood vessels.

### 3.5 HUVECs distribution and migration in vascular scaffold

**FIGURE 7:**
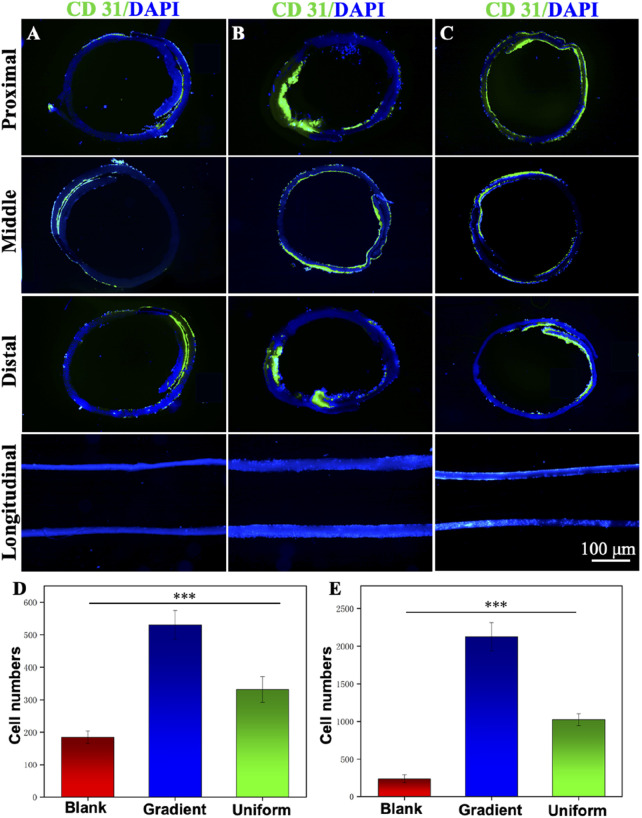
Immunofluorescence staining micrographs showing the cross-sectional and longitudinal observation of the different vascular scaffolds made of **(A)** blank PCL nanofibers, PCL nanofibers coated with **(B)** gradient and **(C)** uniform ECd. Green: CD31; Blue: DAPI. The HUVECs were cultured on the scaffolds for 7 days. **(D)** The number of vascular scaffolds in the longitudinal sections. **(E)** The number of vascular scaffolds in the distal of cross sections. ***P < 0.001 compared to the gradient group.

Supplementary Figure S3 shows an ultra-deep 3D stereomicroscopic image of a bi-directional linear gradient ECd-coated PCL vascular scaffold, which can be viewed as an overall macroscopic view of the scaffold mimicking a normal vascular structure. Figures 7A–C show cross-sectional and longitudinal staining images of blank PCL nanofibers and PCL nanofibers coated with gradient and uniform ECd for distal HUVECs 7 days after inoculation. Immunofluorescence cross-sectional and longitudinal images showed that HUVECs proliferated in the inner lumen and outer surface of the gradient ECd-coated PCL nanofibers (Figure 7B), and some areas showed aggregation of HUVECs with obvious cell proliferation and migration compared with the other two groups. While in the lumen of PCL nanofibers with uniform ECd coating (Figure 7C), some HUVECs grew around the nanofiber scaffolds, and the number of cells were less than that of PCL nanofibers with gradient ECd coating, but in the lumen of blank PCL nanofibers (Figure 7A), the number of HUVECs were less and no cell migration was seen. Figures 7D, E show the statistics of the number of HUVECs in the transverse and longitudinal sections of bi-directional linear gradient ECd-coated PCL vascular scaffolds, and the differences were statistically significant (P < 0.001) when compared with the other groups. The experimental results showed that the guiding fibers and bioactive of the gradient ECd-coated vascular scaffolds prepared by electrospinning techniques facilitated cell attachment. With the increase of ECd content, both the generated ECd gradient and the uniform coatings promoted the migration of HUVECs along the fibers and in the direction of the gradient. The current clinical application of vascular scaffolds cannot mimic the topological structure of natural vascular networks, which is insufficient to induce cell crawling. We aim to address this deficiency by changing the structure and functionalization of biomimetic blood vessels to further enhance the recovery of vascular defects and ruptures.

## 4 Conclusion

Based on the bioregulatory function of bioactive substances derived from cells, we propose that the extracellular matrix obtained from cells has the potential to regenerate injured tissues. In this regard, derivatives of HUVECs were collected using the optimized cell number and culture duration. After freeze-drying, the ECd was electrosprayed and deposited as microparticles on uniaxially aligned PCL nanofibers to explore its potential for rapid endothelialization. Utilizing a self-developed strategy, we achieved uniform or bi-directional coating of ECd on the aligned PCL nanofibers. We demonstrated that the HUVECs grew and migrated in the direction of the fibers and with the increasing ECd content. After rolling the nanofiber mats into the vascular scaffold, we observed improved endothelialization in the lumen and enhanced expression of the vascular marker CD31 in the vascular scaffold using PCL nanofibers coated with gradient ECd. Taken together, this study paves a new path for applying ECd in vascular tissue engineering and promotes the development of cell derivative-based biomaterials for applications related to vascular regeneration and angiogenesis.

We believe this is the first step toward achieving vascular scaffold reconstruction. In our future work, we will further validate the safety and efficacy of vascular scaffolds and explore their potential in treating various diseases, including cardiovascular disease, neuroprosthetics, and tissue engineering. By integrating knowledge and technology from multiple disciplines, such as materials science, cell biology, and biomedical engineering, we will conduct interdisciplinary research to foster innovation and development in vascular scaffold technology.

## Data Availability

The original contributions presented in the study are included in the article/Supplementary Material, further inquiries can be directed to the corresponding authors.
