# Supplementary material for: Gradient coating of extracellular matrix derived from endothelial cells on aligned PCL nanofibers for rapid endothelialization
Source: Front Bioeng Biotechnol. 2025 Jan 8;12:1527046. doi: 10.3389/fbioe.2024.1527046 (PMC11751034; doi:10.3389/fbioe.2024.1527046)
Supplement: Supplementary file 1 [file DataSheet1.docx]

**Supporting Information**

**Gradient coating of extracellular matrix derived from endothelial cells on aligned PCL nanofibers for rapid endothelialization**

Ziyi Zhou ^a,b,‡^, Yijing Lin ^b,‡^, Na Liu ^a^, Yiming Zhang ^b^, Bing Li ^c*^, Yuanfei Wang ^d,*^

^a^Qingdao Medical College, Qingdao University, Qingdao 266071, China.

^b^Department of Plastic, Reconstructive and Cosmetic Surgery, Xinqiao Hospital, Army Medical University, Chongqing 400037, China.

^c^Department of Genetics and Cell Biology, Basic Medical College, Qingdao University, Qingdao 266071, China.

^d^Central Laboratory, Qingdao Stomatological Hospital Affiliated to Qingdao University, Qingdao University, Qingdao 266001, China.

‡ Ziyi Zhou and Yijing Lin contributed equally to this work.

* Corresponding author:

Bing Li (libing_516@qdu.edu.cn)

Yuanfei Wang (zhizunbao19@163.com)


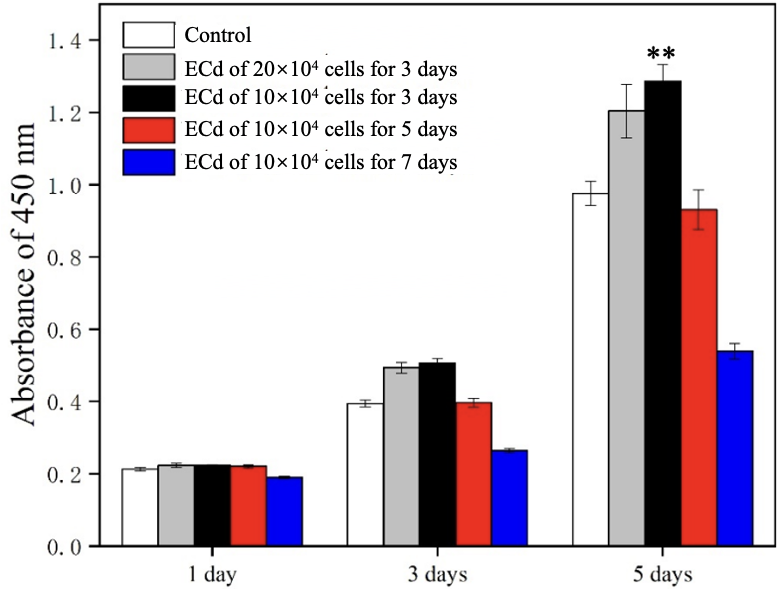


**Fig S1.** Determining the optimal incubation time and cell density for ECd according to the proliferation of HUVECs. ***P* < 0.01 compared with ECd derived from 1 × 10^5^ cells cultured for 3 days.


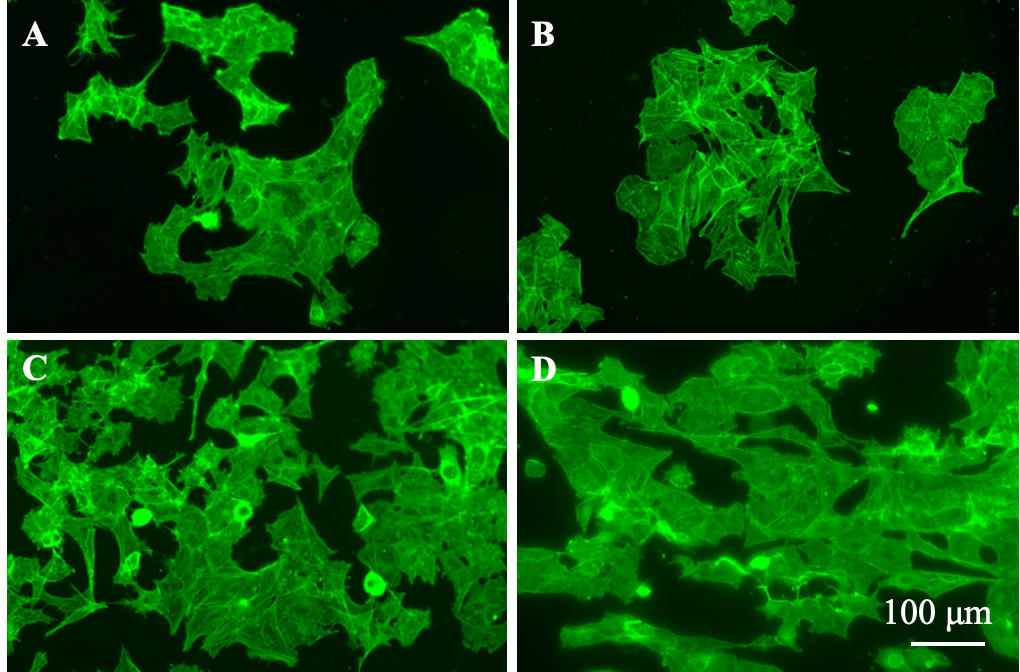


**Fig S2.** Fluorescence micrographs showing the morphologies of HUVECs on (A) control, (B) blank PCL nanofibers, PCL nanofibers coated with (C) gradient, and (D) uniform ECd at 3-day post culture.


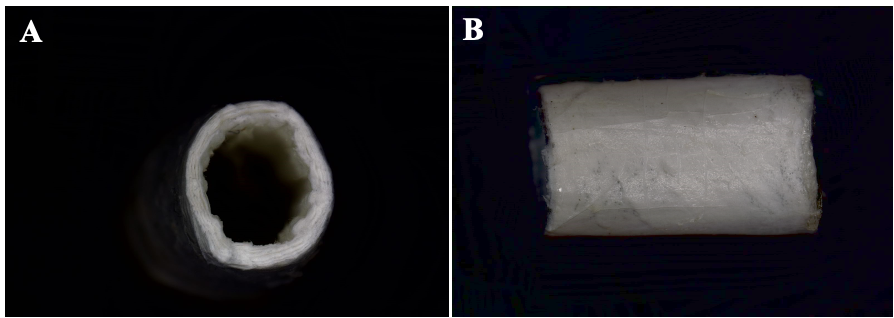


**Fig S3.** Ultra-depth microscopy image of the vascular scaffold made of PCL nanofibers coated with gradient ECd: (A) transverse section (B) longitudinal section.
